# Microstructural Design of Ba_0.5_La_0.5_Co_0.5_Fe_0.5_O_3_ Perovskite Ceramics

**DOI:** 10.3390/ma14164656

**Published:** 2021-08-18

**Authors:** Daria Gierszewska, Iga Szpunar, Francis Oseko, Joanna Pośpiech, Małgorzata Nadolska, Martyna Pieragowska, Karolina Reniecka, Kinga Waniek, Karol Leszczyński, Aleksandra Mielewczyk-Gryń, Maria Gazda, Sebastian Wachowski

**Affiliations:** Institute of Nanotechnology and Materials Engineering, Faculty of Applied Physics and Mathematics, Advanced Materials Centre, Gdańsk University of Technology, 80-233 Gdańsk, Poland; iga.lewandowska@pg.edu.pl (I.S.); avafrancpost@gmail.com (F.O.); pospiechasia@gmail.com (J.P.); malgorzata.nadolska@pg.edu.pl (M.N.); s172601@student.pg.edu.pl (M.P.); s172564@student.pg.edu.pl (K.R.); waniek.kinga@gmail.com (K.W.); s172593@student.pg.edu.pl (K.L.); aleksandra.mielewczyk-gryn@pg.edu.pl (A.M.-G.); maria.gazda@pg.edu.pl (M.G.); sebastian.wachowski@pg.edu.pl (S.W.)

**Keywords:** BLCF, synthesis, microstructure, size of grains, ceramics density, specific area

## Abstract

Ba_0.5_La_0.5_Co_0.5_Fe_0.5_O_3−δ_ was synthesized in the solid-state reaction route. The influence of ball milling parameters (such as milling media size, angular velocity, and time), pelletizing pressure, and annealing parameters on the microstructure was studied. The grain size distribution and density or specific surface area changes were investigated in each approach while the individual parameters were changed. The evaluation of BLCF synthesis parameters enables tailoring the microstructure to various applications. It was observed that with lowering the size of milling balls and increasing the angular velocity the material will be porous and thus more appropriate as electrode material in proton ceramic fuel cell or electrolyzer. An increase of time, balls diameter, and/or angular velocity of milling enables one to densify the material in case of membrane application in, e.g., as a gas sensor. The significant influence on densification has also annealing temperature increase. Applying 1200 °C during annealing leads to dense material, while at 1100 °C shows visible porosity of the product. In this work, we present the results of the BLCF synthesis parameters change allowing the selection of appropriate parameter values depending on the further application as PCCs.

## 1. Introduction

The process of ceramic fabrication is essential for final product properties. For instance, properties such as thermal [1] and electric conductivity [2], gas permeability [3], mechanical strength [3], and the number of catalytically active sites [4] depend on microstructural features, such as surface area, grain size, and grain boundary length. In extreme cases, where the crystal size is reduced to a few nanometres, the material properties become dominated by interfacial effects. All in all, it is important to study not only the material properties themselves but also how the material can be processed, so its microstructure and properties can be tuned for specific application needs. The microstructure often does not receive sufficient attention in the scientific community, leaving the know-how gap stalling the material used in more applied studies or, in the end, in the industrial field. However, the parameters such as grain size distribution play a crucial role in various fields and applications e.g., [5,6]. The case is no different for the field of Proton Conducting Ceramics (PCCs), which aims at the development of materials for high-temperature electrochemical devices, such as gas sensors, fuel cells, or steam electrolyzers [7]. To fabricate such devices, multiple groups of materials must be developed: electrolytes, materials for negatrodes (namely, an anode for fuel cell or cathode for electrolyzer), and positrodes (a cathode for fuel cell or anode for electrolyzer). Yttrium doped barium cerate-zirconate solid solutions (BZCYs) are the most often used ceramics for electrolytes. As for the negatrodes, cermet composites composed of electrolyte material and metal are used. Usually, BZCY and Ni are the materials of choice [8,9].

The positrode performance is what limits current electrochemical devices based on PCCs and much effort was put into their development [10,11,12,13]. Recently, we have focused on the group of BaLnCo_2_O_6_ (Ln—a lanthanide) perovskite compounds that showed promising properties for application as positrodes [14,15,16]. This system exhibits high electrical conductivity in a wide temperature range. The protonic contribution to the electrical conductivity of these perovskites facilitates their work as a positrode material in highly efficient electrochemical devices using protonic transport [16,17,18]. Among them, the materials where Ln is either La or Gd showed the best performance. However, their major drawbacks are high thermal expansion coefficient, small water uptake, and insufficient proton conductivity. A potential solution to this problem might be the partial substitution of cobalt with iron. This may decrease the thermal expansion coefficient (TEC) [19] and improve the water uptake, and therefore the protonic partial conductivity of the compound, since the increase of the average basicity at the B-site is reported to have that effect [11,20]. Therefore, we selected Ba_0.5_La_0.5_Co_0.5_Fe_0.5_O_3_ (BLCF) as the material for this study, as it should both show the benefits of cobaltites, such as high electronic conductivity and catalytic activity, and improved proton conductivity combined with reduced TEC value of ferrites. The main aim of this work was to observe the influence of synthesis parameters on the stoichiometric BLCF microstructure and to determine the mutual dependencies, pointing to the efficiency and effectiveness of the synthesis route. The factors of interest were i.a. ball milling velocity, the size of milling media, time of milling, pelletizing pressure, temperature, and time of sintering. Additionally, basic material characterization concerning the positrode application of this material was performed, such as structure refinement, microstructure observation, grain size analysis, porosity, or specific surface area examination. For the first time, this work describes how the synthesis parameters influence the BLCF microstructure and densification. Understanding the relationship between particular parameters is crucial in a view of future application of this system in fuel cells, electrolyzers, and permeation membranes.

## 2. Experimental Route

### 2.1. Synthesis of Material

**Figure 1:**
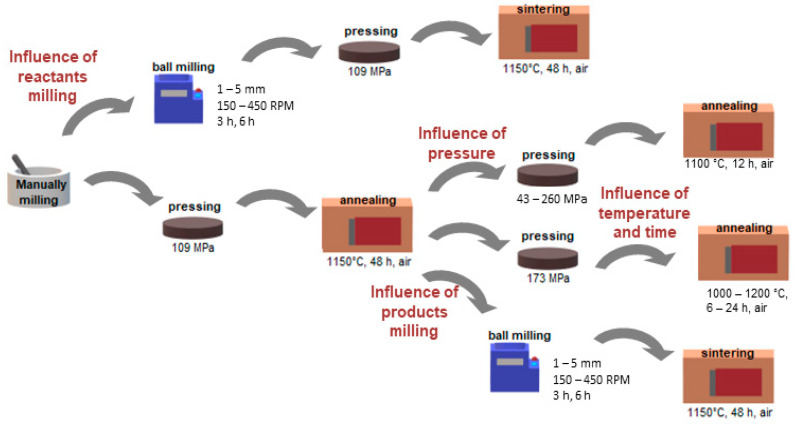
A diagram of the procedures used for the preparation of BLCF ceramics. They include grinding, ball milling, pressing, and annealing processes.

As the study explores the influence of synthesis parameters, multiple routes of synthesis were used and a graphical diagram of the procedures is given in Figure 1. The processing conditions of each sample are summarized in Table 1. All Ba_0.5_La_0.5_Co_0.5_Fe_0.5_O_3_ compounds were synthesized with solid-state reaction method, where the stoichiometric amounts of La_2_O_3_ (pre-annealed for 5 h at 900 °C due to its hygroscopic nature, 99.9% Alfa Aesar, Ward Hill, MA, USA), BaCO_3_ (99.9% Sigma Aldrich, St. Louis, MO, USA), Co_3_O_4_ (99.98% Alfa Aesar), and Fe_2_O_3_ (99.9% Alfa Aesar) were used.

### 2.2. Material Characterisation

Following synthesis and compacting conditions were studied: the conditions of milling of the chemical reactants used for the synthesis of BLCF; the conditions of the sintering step, that is the pressure used for compacting the pellets used for sintering, the temperature, and time of sintering as well as the conditions of milling of the BLCF powder used for pellets preparation.

The first of the effects to study was the effect of ball milling of the chemical reactants on the microstructure. In this case, the reactants were pre-mixed and manually ground in an agate mortar. Then the powders were ball milled with isopropyl alcohol as a medium. Planetary ball mill Fritsch Pulverisette 7 Classic Line (Weimar, Germany) was used. Both milling cups and balls were made of zirconia. The process of ball milling was performed in cycles. Each cycle was 1 h long and consisted of 50 min of milling and a 10 min pause for cooling down. After that, the cycle was repeated. The direction of rotation was changed in each cycle. The varied parameters were milling media diameter varying from 1 to 5 mm, the milling velocity (from 150 to 450 RMP), and the number of milling cycles (between 3 and 6, which corresponds to 3 to 6 h of the total time of milling). The weight ratio between the milling media and the sample was constant in each milling attempt. After milling, the slurries were dried and the obtained powder was pelletized under 109 MPa pressure. The green bodies were annealed at 1150 °C for 48 h in ambient air. 

All of the other effects were studied on BLCF samples synthesized during a similar approach as above, omitting only the planetary milling step. Before further preparation stages, the obtained pellets were crushed and ground in a mortar.

In the pelletizing pressure study, the powders were subsequently uniaxially pressed into pellets under pressure varying from 43 to 260 MPa and then sintered at 1100 °C for 12 h in ambient air.

To evaluate the influence of sintering conditions on the microstructure, the powders were pressed under 173 MPa and then sintered in an air atmosphere. To study the influence of temperature, the annealing was done at one of the following temperatures 1000 °C, 1100 °C, or 1200 °C, while the time of sintering was kept constant at 12 h. For evaluating the effect of time, the specimens were annealed at 1100 °C for the following periods: 6, 12, or 24 h.

To study the effect of ball milling of BLCF powders, the sintered samples after crushing and grinding were balled-milled. Similar to the reactants milling, ball-milling proceeded in isopropyl alcohol while the varied parameters were milling media size varying from 1 to 5 mm, the milling velocity from 150 to 450 RMP, and the number of cycles from 3 to 6. For analysis of ball milling, a mathematical “collision model” [21,22] was used to determine the frequency of collisions, energy, and power of milling by specific parameters. These factors depend on the kinetics and geometry of the mill, as well as the milling balls. Since all the geometric parameters of the mill were fixed, ball depending parameters and coefficients were calculated according to the model [21] and are given in Table 2.

The absolute velocity of a milling ball is given by Equation (1).

(1) $$v_{b}=K_{b}W_{p}R_{p}$$

where $W_{p}$ is a milling angular velocity, $R_{p}$ is a distance between the center of the mill and the center of the vial and it is fixed by the mill model. $K_{b}$ is a geometrical coefficient dependent on a ball diameter and was estimated following [21].

Impact energy is expressed by the relation (2):

(2) $$\Delta E=\frac{1}{2}K_{a}m_{b}v_{b}{}^{2}$$

where $K_{a}$ is a coefficient of balls collision property. Assuming almost perfectly elastic collisions, $K_{a}$ is equal to 1.

The collision frequency f is needed for the calculations of milling power and is given by Equation (3).

(3) $$f=KkW_{p}N_{b}=K_{v}W_{p}N_{b}$$

K is a factor expressing the time of energy dissipation and it depends on the ball diameter. k is a kinetic parameter of the mill. Both parameters were established based on the [21] and the product of them is later referred to as constant $K_{v}$.

Power of milling can be written as follows:

(4) $$P_{cal}=\varphi N_{b}\Delta Ef$$

where φ is the parameter of vial filling that can be written as:

(5) $$\varphi =1-{\left(\frac{N_{b}}{N_{b\;max}}\right)}^{\varepsilon }$$

ε is a parameter that depends on a ball diameter. φ is equal to 0 for a filled vial (no movement of a ball possible) and 1 when it is empty. Based on the studies, Burgio et al. [21] assumed that until one-third of the vial is not filled, φ is close to 1. In this paper, calculations were determined directly, yielding a value of 0.99 for the case of each experiment. 

The structural characterization and phase analysis were carried out by X-ray Diffraction (XRD) using a Phillips X’Pert Pro X-ray diffractometer (Almelo, The Netherlands) with Cu*K*_α_ radiation. Powder X-ray diffraction data were collected by step scanning over in the range 10–125° in 2θ with an increment of 0.02°. The X-ray pattern data of samples prepared in different route are included in Supplementary Material. The unit cell parameters of the as-synthesized BLCF were determined with the use of Rietveld refinement using GSAS-II software (Argonne National Laboratory, Argonne, IL, USA) [23]. The initial model used for analysis was cubic perovskite structure (space group $Pm\bar{3}$m, the number of the space group: 221) with ID number 7033669 from COD PDF, 2015 (Crystallography Open Database Powder Diffraction File) [24]. The A-site in the model was occupied by Ba and La, each with 0.5 occupancies. Similarly, the B-site was set to be occupied with Co and Fe with 0.5 occupancies. In the analysis, the occupancies of cations were fitted parameters. Since the XRD is not sensitive towards lighter elements the O occupancy had to be fixed.

The average oxidation state of the B-site elements (cobalt and iron) was determined using iodometric titration. Small amounts of sample (16.8 mg) and potassium iodide (about 0.2 g) were dissolved in 2 M HCl with a magnetic stirrer. The sodium thiosulfate was titrated until the solution became transparent. Its volume was used for the calculation of average oxidation state and oxygen non-stoichiometry. The titration was carried out in inert conditions. The average B-site oxidation state was used to calculate the oxygen non-stoichiometry and then used as a fixed point for O occupancy in the refinement.

The microstructure of each specimen was evaluated with the use of Scanning Electron Microscopy (SEM). FEI Quanta FEG 250 microscope (FEI Company, Hillsboro, Oregon, USA) with ETD detector working in secondary electron (SE) mode was used for analysis. The imaging was performed in high vacuum mode. The images were analyzed using ImageJ software (1.8.0, National Institute of Health, Rockville Pike, MA, USA) to determine the grain size distributions. Additionally, the average grain size and the standard deviation of the mean were calculated.

For each bulk specimen, the density was determined, while for the powder samples—the specific surface area was measured. The density of pellets was measured using the Archimedes method. The samples were weighed in air and inserted in a beaker filled with kerosene. Then, they were placed in a vacuum desiccator to enable removing the air from the pores. Using the Archimedes set mounted on laboratory balance, samples were weighed in air and in a beaker with kerosene to calculate the densities. The specific surface area was measured by the BET (Brunauer–Emmett–Teller) method on powder samples using Quantachrome NOVAtouch NT-LX-1 gas sorption analyser (Quantachrome Instruments, Boynton Beach, FL, USA). Before the measurements powders were degassed at 300 °C for 3 h under vacuum. The surface area value was determined from the nitrogen desorption isotherm in a relative pressure range from 0.1 to 0.2.

The electrical properties of the optimized pellet have been measured by the means of the 4-wire DC probe method using KEYSIGHT 34790A (Keysight Technologies, Pulau Pinang, Malaysia). Rectangular bars were cut from the densest specimen. Silver stripe electrodes have been applied to it by brush painting. The electrical conductivity has been measured in the temperature range 300–800 °C with a step of 50 °C. During each step, voltage drop was monitored during and after temperature change until the equilibrium was reached. Only then the measurement of conductivity was performed. The measurements were done in two atmospheres: dry ($p_{O_{2}}=0.195\;atm.$ and $p_{H_{2}O}=10^{-5}\;atm.$) and humidified ($p_{O_{2}}=0.195\;atm.$ and $p_{H_{2}O}=0.023\;atm.$) air. The gas mixtures were obtained with a home-built gas mixer by balancing O_2_ and N_2_ gases which then flew through either drying or wetting stages. A wetting stage consists of two Dreschel bubblers connected in series, where the first bottle was filled solely with deionized water and the second bubbler was filled with a supersaturated solution of deionized water with KBr (99.0%, Acros Organics, Waltham, MA, USA). A drying stage consists of two tubes filled with drying agents connected in series. The first drying agent is silica gel and the second is P2O5 (98%, Alfa Aesar). Gas partial pressures were calculated with ProgasMix FC software (v 0.7.1, NORECS, Oslo, Norway) [25].

## 3. Results and Discussion

### 3.1. Structural Analysis

**Figure 2:**
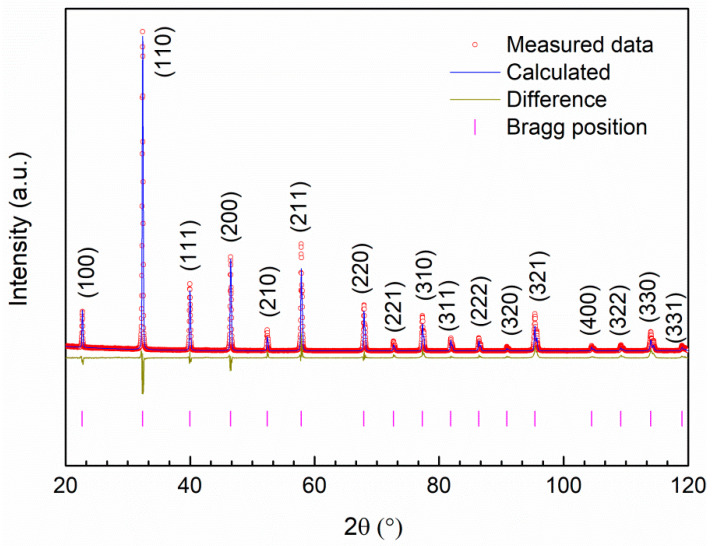
The Rietveld refinement profile of the Ba_0.5_La_0.5_Co_0.5_Fe_0.5_O_3−δ_ powder (R_w_ = 16.77%, Re = 12.14%). The data points are denoted by circles, the refined pattern is a solid line. Below the refined pattern, a difference plot is shown. Bragg positions of the cubic perovskite structure phase are presented by vertical bars at the bottom.

All studied powders were single-phase cubic perovskites with $Pm\bar{3}$m space group. The results of Ba_0.5_La_0.5_Co_0.5_Fe_0.5_O_3−δ_ structural refinement are presented in Figure 2. The lattice parameter is 3.9033(1) Å, which is in agreement with previously reported data [26] and the calculated theoretical density is equal to 6.79 g·cm^−3^. The estimation of the oxidation state of ferrite-cobaltite perovskite is available under the assumption that the value refers to the average sum of oxidation state for Fe and Co cations, and their contribution is not known [27], therefore, we calculated the average oxidation state of these two cations and by this oxygen non-stoichiometry. The refinement and titration results are summarized in Table 3.

### 3.2. The Effect of Ball Milling of Precursors on the Microstructure of BLCF Ceramics

**Figure 3:**
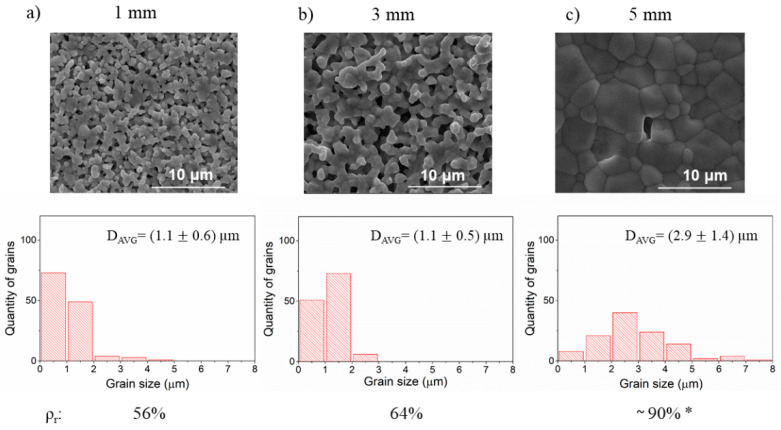
The SEM images of the BLCF ceramics obtained using balls of different diameters for the stage of reactants milling. Underneath each micrograph, an individual grain size distribution with corresponding relative density value (ρ_r_) is shown. (**a**–**c**). * The sample cracked during the density measurement, thus the result should be treated as an estimation.

The results of the effects of the size of milling media balls on the microstructure of the ceramic are summarized in Figure 3. The other parameters of the milling process were kept constant i.e., angular velocity was 300 RPM and three cycles of milling were used (3 h). Both SEM images and density measurements clearly show that density as well as the grain size of ceramic increases with the increasing diameter of milling balls used for milling. Interestingly, using the balls of 5 mm diameter results in a significant densification rise. Further inspection of micrographs shows that the average size of grains and the width of the size distribution of grains increase as the size of milling media increases. This growth corresponds also to the increase of ceramic density.

Since all parameters of the milling except the milling media size were kept constant, the major differences between the milling procedures were the frequency of collisions and impact energy of each collision. As the size of the milling ball increases the number of balls used in the process decreases and therefore the frequency of collisions is declining. At the same time, the energy of each collision increases with the ball size, because the larger ball has a larger mass, and the impact energy is proportional to it. Both impact frequency and energy were calculated using the collision model. The calculated values of impact energy, power of milling, frequency, and other important parameters for all milling processes evaluated in this study are gathered in Table 4.

For the given parameters, as the diameter of the balls increases from 1 to 3 and to 5 mm, the frequency decreases from 4785 hit·s^−1^ via 269 hit· s^−1^ to 57 hit· s^−1^, respectively. The respective impact energies are $1.4\times 10^{-5}\;J\cdot {\mathrm{hit}}^{-1}$, $2.2\times 10^{-4}\;J\cdot {\mathrm{hit}}^{-1}$, and $9\times 10^{-4}\;J\cdot {\mathrm{hit}}^{-1}$. Therefore, the smaller diameter of balls leads to a higher frequency of hits of relatively low energy. It results in a formation of a large number of small grains of similar size in the resulting ceramics. On the other hand, using the 5 mm balls corresponds to a low frequency of hits of high energy. As a result, both the average grain size and the distribution of grain sizes increases. The high energy of each impact might activate the surface of reactant grains enhancing in this way their fast growth and higher sinterability. The high density of these ceramics is most probably related to both the above factors. It was observed before that the densification rate of ceramics with a broad size distribution is higher at the early stages of sintering [28].

**Figure 4:**
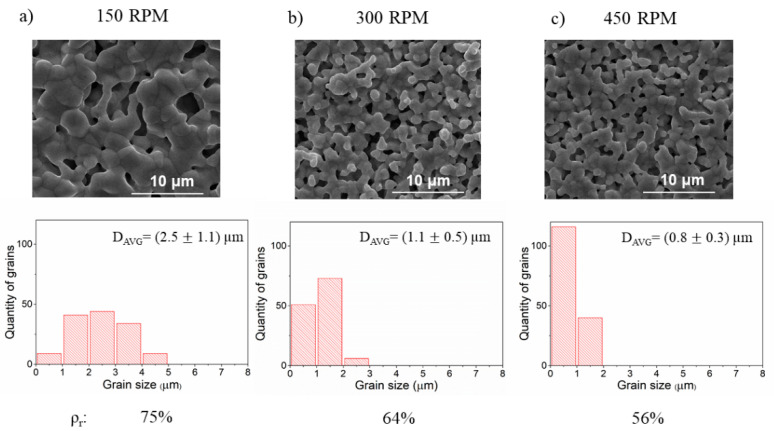
The SEM images of the BLCF pellets obtained during reactants milling with different angular velocities. Underneath each micrograph, an individual grain size distribution with corresponding relative density value (ρ_r_) is shown. (**a**–**c**).

The SEM analysis results demonstrating how the microstructure changes with the angular velocity of reactants milling are shown in Figure 4. In this case, the highest relative density was obtained for the lowest angular velocity. It is noticeable that grain size distribution, in this case, is also the widest. With increasing velocity, the density decreases and the grains are smaller, showing a narrow grain size distribution. As the angular velocity increases all milling parameters increase: frequency of collisions, impact energy, power of milling, and total energy. Comparison of the obtained results for varying the size of milling media and the angular velocity leads to a conclusion that higher collision frequency means higher porosity and smaller grain size. 

**Figure 5:**
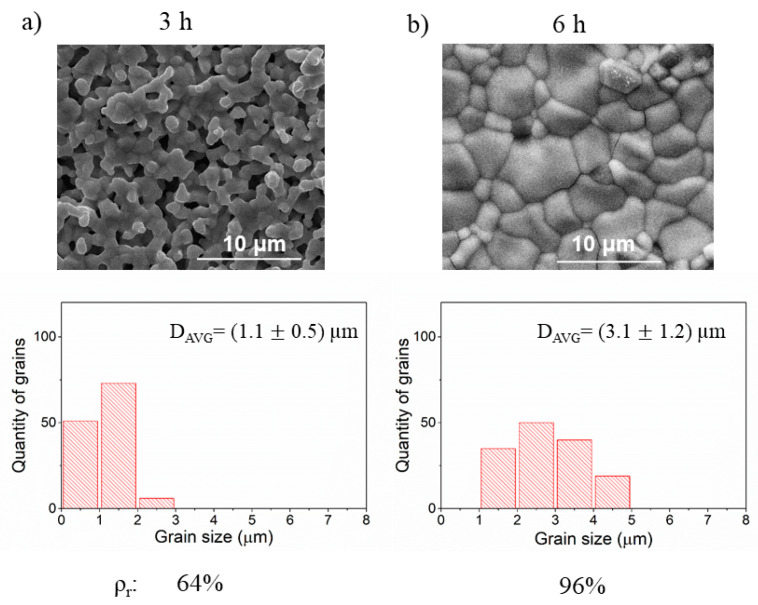
The SEM images of the BLCF pellets obtained during a different time of the reactants milling. Underneath each micrograph and individual grain size distribution with corresponding relative density value (ρ_r_) is shown. (**a**,**b**).

In the last test, the number of milling cycles, and therefore the total time of milling, has been doubled. The results of the SEM evaluation are given in Figure 5. Extended milling time significantly increases relative density, which results in the highest value of all three milling parameters changes. It also enhances the growth of the grain. In this case, also like in the previous experiments, concerning media size and velocity milling changes, the densest sample shows the widest grain size distribution. One possible explanation could be that a long time leads to efficiently more energy transferred to the specimen, especially, if pauses between milling cycles are not long enough for cooling of the powder being milled. In this way, the heat accumulates throughout the process and might cause chemical reactions between the compounds. Such by-products of milling might be active chemically and thus they might promote sintering during thermal annealing.

### 3.3. Influence of BLCF Pelletizing Pressure on the Ceramic Microstructure

**Figure 6:**
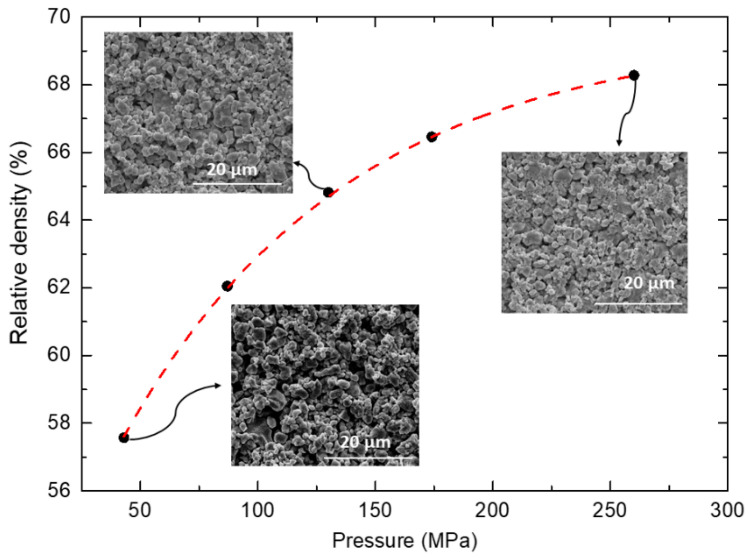
The relative density (%) as a function of applied pressure (MPa). The insets show SEM images of the surface of selected samples.

The influence of pressure on the microstructure was investigated by changing the value of applied pressure during pelletizing process. The relationship between relative density and applied pressure accompanied with SEM micrographs of each pellet is shown in Figure 6. The results show that with increasing pressure, the density increases. The difference between density achieved for 43 MPa and 87 MPa force values reaches nearly 5%, while further increase above 87 MPa increases the density only about 2%. It seems that above 260 MPa, the curve tends to saturate and further increase of the pressure may not lead to significantly higher density. The phenomenon of saturation of compaction is common for both ceramic and metal powders e.g., [29,30,31]. There are several models in the literature for analyzing the densification of different powders. Numerous mechanisms can be considered in the densification process, including diffusional redistribution of matter, plastic flow, and power-law creep [31,32]. However, each model is based on empirical data. Therefore, one has to examine multiple pressure values to find the optimum pressing pressure for a particular powder. The SEM images of the surface of selected pellets show similar characteristics and do not indicate distinct microstructural changes. This suggests that applied pressure influences solely the density value, compaction of green bodies, not the sample’s microstructural features like grain size.

### 3.4. Influence of Time and Temperature of Annealing on Sintering of BLCF

**Figure 7:**
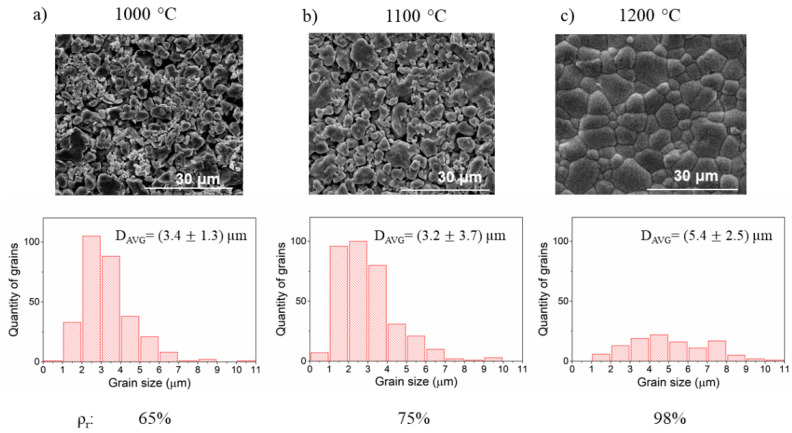
The SEM images, grain size distribution, and relative density ρ_r_ of ceramics annealed at (**a**) 1000 °C, (**b**) 1100 °C, and (**c**) 1200 °C.

The influence of the annealing parameters was investigated by varying two parameters: temperature and time of sintering. The results of SEM analysis of the samples annealed at 1000 °C and 1200 °C for 12 h are presented in Figure 7. It can be seen that significant densification occurs at 1200 °C, while at lower temperatures the relative density is lower. However, in the more porous samples, the grains have a more homogeneous size distribution. It is seen a tendency that with increasing annealing temperature, the grain size distribution extended and the sample densified.

**Figure 8:**
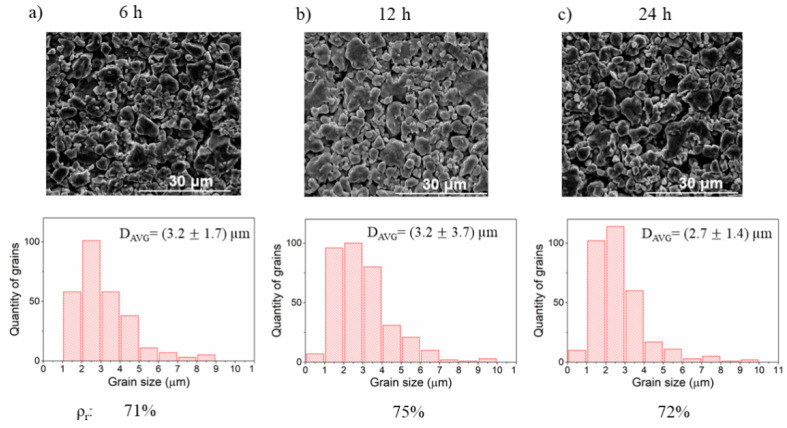
The SEM images, grain size distribution, and relative density ρ_r_ of ceramics annealed at 1100 °C for (**a**) 6 h, (**b**) 12 h, and (**c**) 24 h.

In the second approach, the samples were annealed for 6 h, 12 h, and 24 h at the same temperature—1100 °C. The results are summarized in Figure 8 and show relatively small changes in the relative density of samples. Interestingly, for the longest time of annealing, the sample has not achieved the highest density. However, the maximum change of relative density is 4%, which is close to the uncertainty of the measurement (above 2%). This indicates that the densification occurs in a time shorter than 6 h and a longer treatment time does not lead to further densification. In such a case the observed differences in density come merely from statistical variations in parameters of the synthesis process, such as slight changes of pressure during pelletizing or random formation of microcracks during annealing.

### 3.5. The Effect of Ball Milling of BLCF Powders on the Grain Size Distribution and Specific Surface Area

In view of a strong influence of the milling conditions of reactants on the density of ceramics, in this part, we study how the ball milling of the BLCF powders before the final sintering step influences the ceramic density and microstructure. The approach was analogous to the investigation of reactants milling. The analyzed parameters were the size of the medium, angular velocity, and time. After milling the microstructure of the BLCF powder was analyzed by SEM and the surface area of each milled powder was determined. The surface area of the reference BLCF sample, which was only crushed in a mortar and was not subjected to ball milling, was equal to 0.48 m^2^·g^−1^.

**Figure 9:**
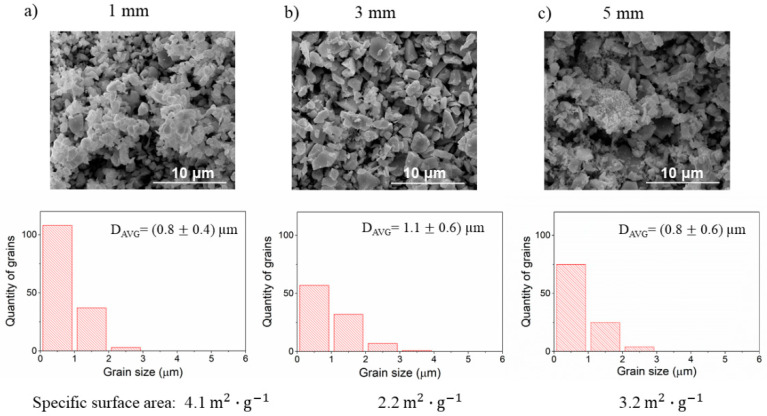
The SEM images with grain size analysis and specific surface area of the BLCF powders milled with (**a**) 1 mm, (**b**) 3 mm and (**c**) 5 mm balls.

**Figure 10:**
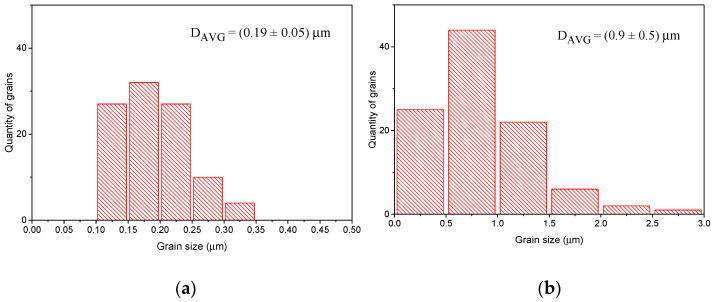
Grain size distribution of grains (**a**) below and (**b**) above 0.5 μm size for BLCF milled with 5 mm diameter balls.

In the first step, the ball diameter was changed from 1 to 3 and to 5 mm. The angular velocity and time of milling were kept constant with values 300 RPM and 3 h, respectively. The results of SEM analysis along with the specific surface measured by BET are shown in Figure 9. After the ball milling, the specific surface area increased in comparison to the reference sample by one order of magnitude. The use of the smallest media leads to the finest grain fragmentation and the highest specific surface area. The use of the 3 mm balls leads to the lower area and larger grains. Interestingly, for 5 mm balls, the surface increases again. Similarly, the grain size distribution indicates that the size distribution in this specimen seems to be in between the specimens milled by 1 mm and 3 mm balls. These results required a closer inspection of the SEM micrograph of the 5 mm balls milled sample. In Figure 10, it is shown that the powder consists of two fractions with significantly different average sizes: one fraction with submicron size and the other with the size of a few microns. It can be seen that a high number of grains smaller than 350 nm, up to roughly 25% of the total number of grains, is present in the BLCF powder. These grains have a small contribution to the total volume of powder, but significantly increase the specific surface area, which is reflected in the obtained BET results. From the milling energy analysis presented in Table 3, one may conclude that the energy of each impact during milling with 5 mm balls might lead to high fragmentation upon collision, but low frequency of collisions caused that most of the grains have not been milled. It should be noted that the collision model used for milling energy analysis does not consider the powder properties and therefore the energy calculated should be the same both for the milling of the reactants and for the products of BLCF synthesis.

**Figure 11:**
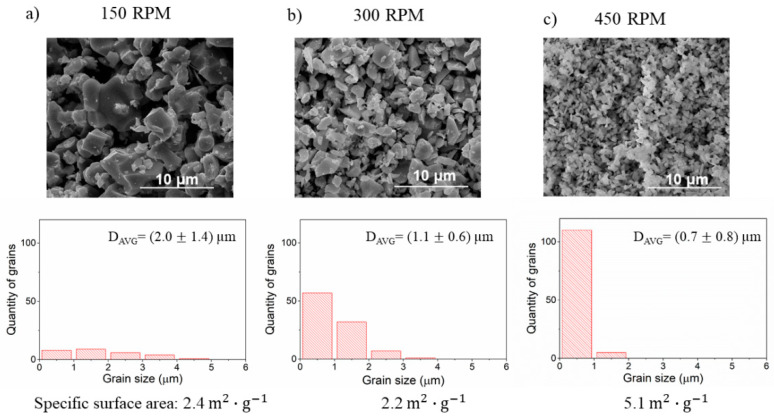
The SEM images, grain size analysis, and specific surface area of the BLCF powders milled with angular velocity (**a**) 150 RPM, (**b**) 300 RPM, and (**c**) 450 RPM.

The investigation of how the angular velocity affects the powder properties was carried out using 150 RPM, 300 RPM, and 450 RPM. The results are summarized in Figure 11. The grain size is reduced with the increasing speed of milling. While increasing angular velocity from 150 to 300 RPM the grain size distribution shifted towards lower values but the specific surface area slightly decreased, which is within the margin of the uncertainty. The most significant difference in grains fragmentation occurs while speed is increased from 300 RPM to 450 RPM. The 450 RPM value ensures the most effective process of fragmentation with the high quantity of small grains (below 1 μm) resulting in the highest specific surface area.

**Figure 12:**
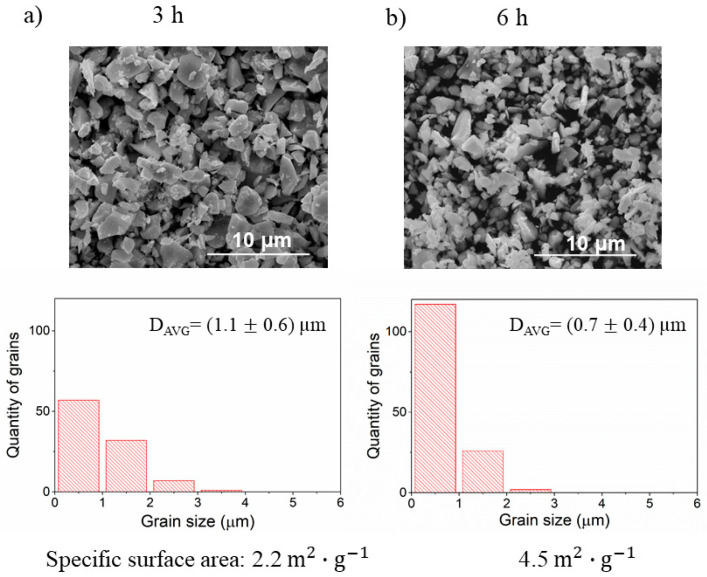
The SEM images, grain size analysis, and specific surface area of the powders milled for (**a**) 3 h and (**b**) 6 h.

The effect of different times of milling powder is presented in Figure 12. An increase of time from 3 to 6 h roughly doubles the specific surface area from 2.2 m^2^·g^−1^ to 4.5 m^2^·g^−1^. Similarly, the grain size distribution shows that powder fragmentation increases with the time of milling, and grains with sizes below 1 μm dominate the specimen milled for 6 h.

### 3.6. Conductivity of BLCF

**Figure 13:**
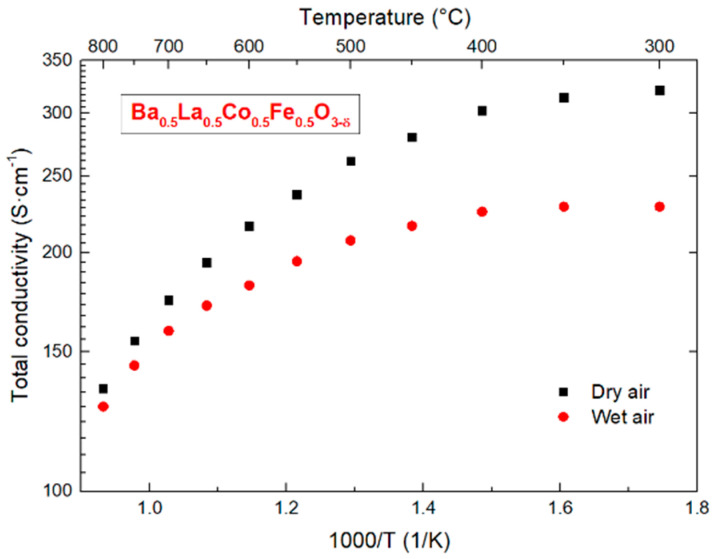
Temperature dependence of total conductivity for Ba_0.5_La_0.5_Co_0.5_Fe_0.5_O_3−δ_ recorded in dry and humidified air.

In order to evaluate the possible use of BLCF as a mixed conductor for electrodes or membrane, a preliminary conductivity study has been performed by the 4-point DC method. The relation of total conductivity vs reciprocal temperature is depicted in Figure 13. It can be seen that the conductivity of the compound is high (~120–300 S·cm^−1^) in both atmospheres in the whole temperature range. This is very promising for the potential application. Interestingly, a difference between total conductivity values for dry and humidified conditions is observed. This is not typical in materials such as ferrites and cobaltites which are dominated by electronic charge carriers. In electron or hole-dominated materials changes in ionic conductivity are often not visible in the total conductivity measurement. However, assuming that BLCF total conductivity is dominated by electron holes and that material can take up protons from humid atmosphere one might derive a typical hydrogenation reaction which describes the situation upon exposure of BLCF to water vapor:

(6) $$H_{2}O_{\left(g\right)}+2O_{O}^{x}+2h^{\cdot }=2OH_{O}^{\cdot }+\frac{1}{2}O_{2\left(g\right)}$$

where $O_{O}^{x}$ denote oxygen ions in the BLCF lattice, $h^{\cdot }$ in an electron-hole and $OH_{O}^{\cdot }$ a protonic defect, while $H_{2}O_{\left(g\right)}$ and $O_{2\left(g\right)}$ represent gas molecules of water vapor and oxygen, respectively. If material conductivity is dominated by electron holes and protons are formed via hydrogenation then in wet atmospheres material would have slightly lower conductivity because the concentration of holes reduces each time a protonic defect is formed. Certainly more studies are required to prove such a hypothesis, but several factors support it. First of all, water uptake was observed in many Ba-based cobaltites and ferrites with perovskites structures [16,20,33,34]. Most importantly, proton formation was observed in Ba_0.5_La_0.5_CoO_3−δ_ [16], which is a Fe-free equivalent of BLCF. Moreover, from studies of similar compounds, such as Ba_0.5_Sr_0.5_Co_1−x_Fe_x_O_3−δ_ and Ba Co_1−x_Fe_x_O_3−δ_, we know that increasing Fe content increases the concentration of formed proton defects [20]. Therefore, it should be expected that even more proton defects are formed in BLCF than in Ba_0.5_La_0.5_CoO_3−δ_. Another argument for proton defect formation in the material is that the difference in conductivity between dry and wet air is diminishing with a temperature increase. Such a phenomenon is typical of protonic ceramics and examples can be shown not only for ferrites and cobaltites [20,35] but also for other systems such as barium zirconate-cerate [36] and lanthanum orthoniobates [37]. This indirectly confirms that proton defects should be expected to form in BLCF in humid atmospheres. Moreover, ferrites and cobaltites conductivity is expected to be dominated by electron-holes in oxidizing atmospheres which was shown in numerous publications [14,25,38,39,40]. Therefore, it can be stated that both data on electron-hole conductivity and protonic defect formation are consistent with the results shown here and their interpretation. Finally, this leads to a conclusion that BLCF is a mixed proton-electron conductor, which makes it a suitable candidate material for proton ceramic electrochemical cells, although more studies are required to describe the partial contributions of various charged carriers to total conductivity.

## 4. Conclusions

BaLaCoFeO_6_ ceramics microstructure can be tailored by altering the synthesis parameters. Ball milling of reactants before the solid-state synthesis can lead to the formation of porous ceramic with the submicron grain size, if small milling media, such as 1 mm diameter balls, or high angular velocity, for instance, 450 RPM, are used in the milling process. On the other hand, large-diameter balls or prolonged milling times lead to a fully densified product. In that way, one may only by choosing reactant milling conditions to fabricate porous ceramics for electrodes for fuel cells or electrolyzers, or develop a dense material for other applications, such as permeation membranes.

The effect of pelletizing pressure on ceramic density showed that in the range of 50–150 MPa the density of the final product linearly depends on the pelletizing pressure, while above 200 MPa further increase of pressure does not lead to much denser product. Varying the temperature of annealing showed that 1200 °C is enough to fully densify the product while 1000 °C and 1100 °C leads to porous ceramics with 35% and 25% porosity, respectively.

Altering the time of annealing revealed that times longer than 6 h do not lead to further increase of density, so 6 h is sufficient time for sintering.

Lastly, the milling of products showed that to obtain high product fragmentation one should use high angular velocity (e.g., 450 RPM), small milling media (1 mm diameter balls), or a long time of milling (6 h). Applying such conditions can lead to a tenfold increase of specific area, in comparison to reference not milled powder and formation of powder dominated by submicron grains.

Preliminary transport measurements data shows that BLCF is a mixed electronic-protonic conductor and promising material for applications is protonic ceramic electrochemical cells.

## Figures and Tables

**Table 1:** List of samples with particular synthesis parameters values used in this study.

Proces	Sample ID	Changed Parameters			Additional Information
		Size of Media (mm)	Velocity (RPM)	Time (h)	
Precursors milling	PM1	1	constant 300	constant 3	In each preparation, the mass of media was constant and equal to 8.5 g. The mass of precursors was about 2 g. Isopropanol was used as the solution during ball milling.
	PM2	3			
	PM3	5			
	PM4	constant 3	150	constant 3	
	PM2		300		
	PM5		450		
	PM2	constant 3	constant 300	3	
	PM6			6	
Pelletizing pressure		Pressure (MPa)			All samples during preparation were manually ground, pressed under 109 MPa into pellets, and sintered at 1150 °C for 48 h in the air before examination of how pelletizing pressure affects the microstructure.
	PP1	43			
	PP2	87			
	PP3	130			
	PP4	174			
	PP5	260			
Annealing		Temperature (°C)		Time (h)	Each sample was manually ground, pressed under 109 MPa, sintered, and reground before pressing under 173 MPa. During further annealing, different temperatures and time values were applied.
	A1	1000		constant 12	
	A2	1100			
	A3	1200			
	A4	constant 1100		6	
	A2			12	
	A5			24	
BLCF powders milling		Size of media (mm)	Velocity (RPM)	Time (h)	In each preparation, the mass of media equaled 8.5 g. The mass of precursors was about 2 g. Isopropanol was used as the solution during ball milling.
	BM1	1	constant 300	constant 3	
	BM2	3			
	BM3	5			
	BM4	constant 3	150	constant 3	
	BM2		300		
	BM5		450		
	BM2	constant 3	constant 300	3	
	BM6			6	

**Table 2:** Parameters and factors of milling balls calculated according to [21,22], *K_a_*—constant describing elastic and inelastic properties of collision, *K_b_*—ball-size related geometrical coefficient, *N*—the number of balls, *N_max_*—number of balls for maximum fulfillment of the vial, φ—fulfillment factor.

Balls Diameter (mm)	$K_{a}$	$K_{b}$	N	$N_{max}$	φ
1	1	1.06	1700	9863	0.99
3	1	1.01	100	543	0.99
5	1	0.98	22	115	0.99

**Table 3:** Structural parameters obtained from the Rietveld refinement of the Ba_0.5_La_0.5_Co_0.5_Fe_0.5_O_3−δ_ pattern along with iodometric titration results.

Atom	a (Å)	Wyckoff Position	Position			Occupancy	B_AVG_	δ
			x	y	z			
Ba	3.9033(1)	1a	0	0	0	0.49	3.42	0.04
La		1a	0	0	0	0.51		
Co		1b	½	½	½	0.5		
Fe		1b	½	½	½	0.5		
O		1c	½	½	0	0.96		

**Table 4:** Basic parameters of milling and characteristic energies of milling calculated according to [21,22]. *D*—diameter of milling ball, *m*—the mass of the ball, *N*—the number of balls, *ω*—angular velocity of milling disc, *E_imp_*—impact energy, *f*—frequency of hits, *P*—the power of milling, *t_tot_*—the total time when the disc was rotating during the milling process, *E_tot_*—total energy transferred during the milling.

Change	*D* (mm)	*m* (g)	*N*	*Ω* (RPM)	*E_imp_* $\left(10^{-4}\;J\cdot {\mathrm{hit}}^{-1}\right)$	*f* $\left(\mathrm{hit}\cdot s^{-1}\right)$	P^*^ (10^−2^ W)	*t_tot_* (min)	E_tot_^*^ (J)
diameter	1	0.005	1700	300	0.1	4785	6.4	150	580
	3	0.087	100	300	2.2	269	5.7	150	516
	5	0.387	22	300	9.0	57	5.1	150	458
velocity	3	0.087	100	150	0.5	134	0.7	150	65
	3	0.087	100	300	2.2	269	5.7	150	516
	3	0.087	100	450	4.9	403	19.3	150	1740
time	3	0.087	100	300	2.2	269	5.7	150	516
	3	0.087	100	300	2.2	269	5.7	300	1030

* The values are rough estimates due to uncertainties connected with choosing proper constant factors for calculations—they can be used to compare the differences within the study, but they are not absolute values and should not be used to compare with other studies.

## Data Availability

Not applicable.

## Supplementary Materials

The following are available online at https://www.mdpi.com/article/10.3390/ma14164656/s1, Figure S1: The XRD pattern of BLCF samples obtained during milling precursors with different ball milling parameters. Figure S2: The XRD data of BLCF synthesized sample after pelletizing pressure change investigation. Figure S3: The XRD data of BLCF synthesized during investigation effect of annealing parameters change. and Figure S4: The XRD data of BLCF synthesized during investigation effect of ball milling of powders.
