# Supplementary material for: Microstructural Design of Ba0.5La0.5Co0.5Fe0.5O3 Perovskite Ceramics
Source: Materials (Basel). 2021 Aug 18;14(16):4656. doi: 10.3390/ma14164656 (PMC8398124; doi:10.3390/ma14164656)
Supplement: Supplementary file 1 [file materials-14-04656-s001.zip › materials-1326418-supplementary.pdf]

Supplementary Information

Microstructural design of $Ba_{0.5}La_{0.5}Co_{0.5}Fe_{0.5}O_3$ perovskite ceramics

Daria Gierszewska, Iga Szpunar, Francis Oseko, Joanna Pośpiech, Małgorzata Nadolska, Martyna Pieragowska, Karolina Reniecka, Kinga Waniek, Karol Leszczyński, Aleksandra Mielewczyk-Gryń, Maria Gazda and Sebastian Wachowski

Institute of Nanotechnology and Materials Engineering, Faculty of Applied Physics and Mathematics, and Advanced Materials Centre Gdańsk University of Technology, Gdańsk, Poland

The XRD pattern were measured and the results are presented for each investigated effect separately in the FIGURE S1 – FIGURE S4. In FIGURE S1 as-synthesized samples prepared during different ball milling parameters of precursors was compared.

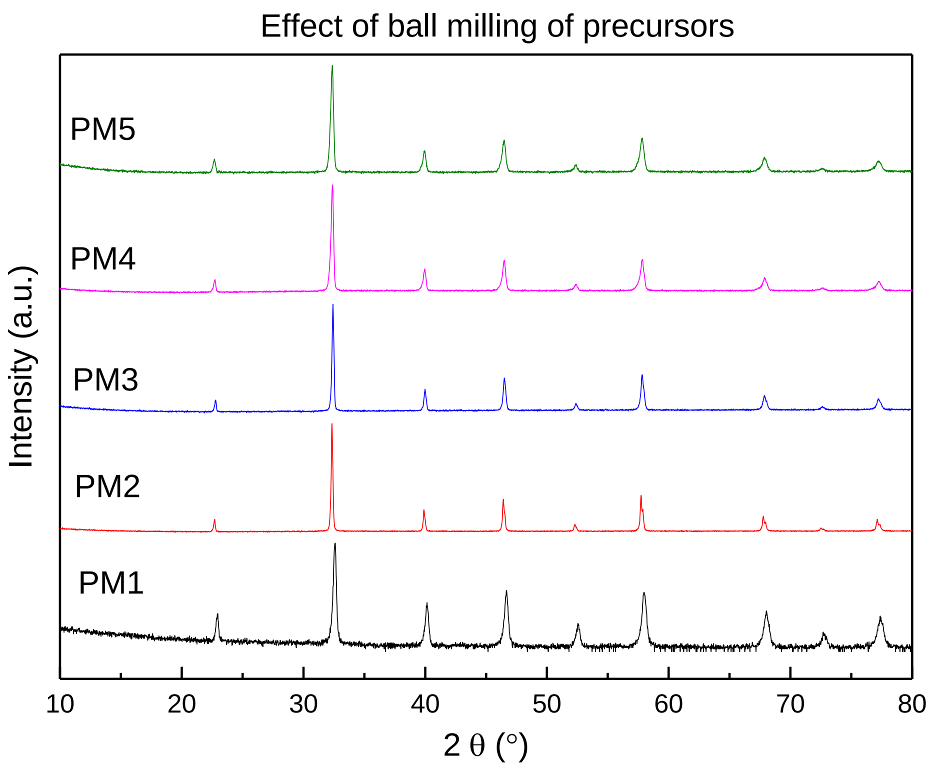

FIGURE S1 The XRD pattern of BLCF samples obtained during milling precursors with different ball milling parameters.

In FIGURE S2 was shown as-synthesized sample prepared to investigate the effect of pelletizing pressure on BLCF microstructure.

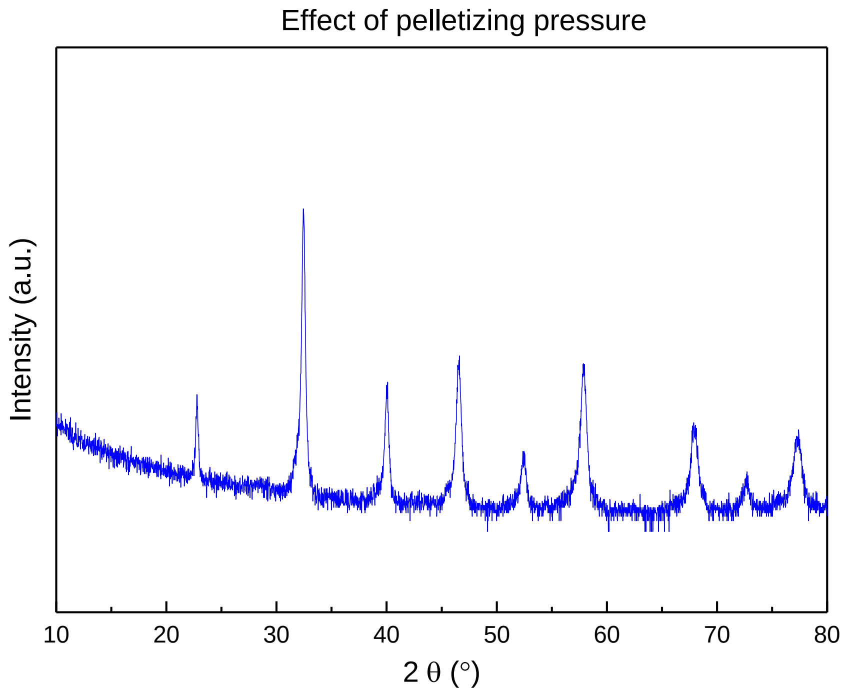

FIGURE S2 The XRD data of BLCF synthesized sample after pelletizing pressure change investigation.

In FIGURE S3 is presented as-synthesized BLCF sample prepared to investigate the effect of change the temperature and time of annealing on microstructure.

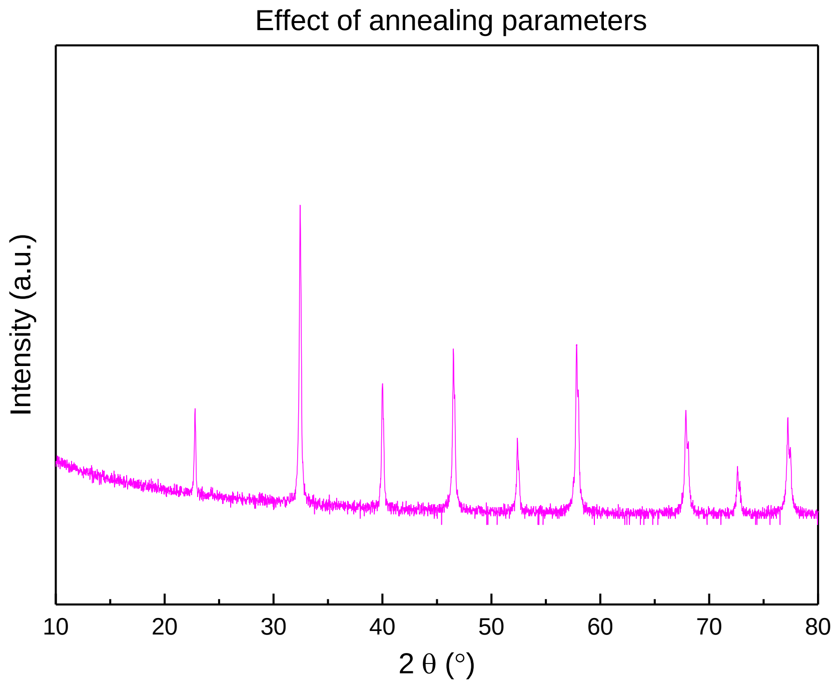

FIGURE S3 The XRD data of BLCF synthesized during investigation effect of annealing parameters change.

In FIGURE S4 is presented as-synthesized sample prepared during investigation of how the BLCF powder ball milling affects the microstructure.

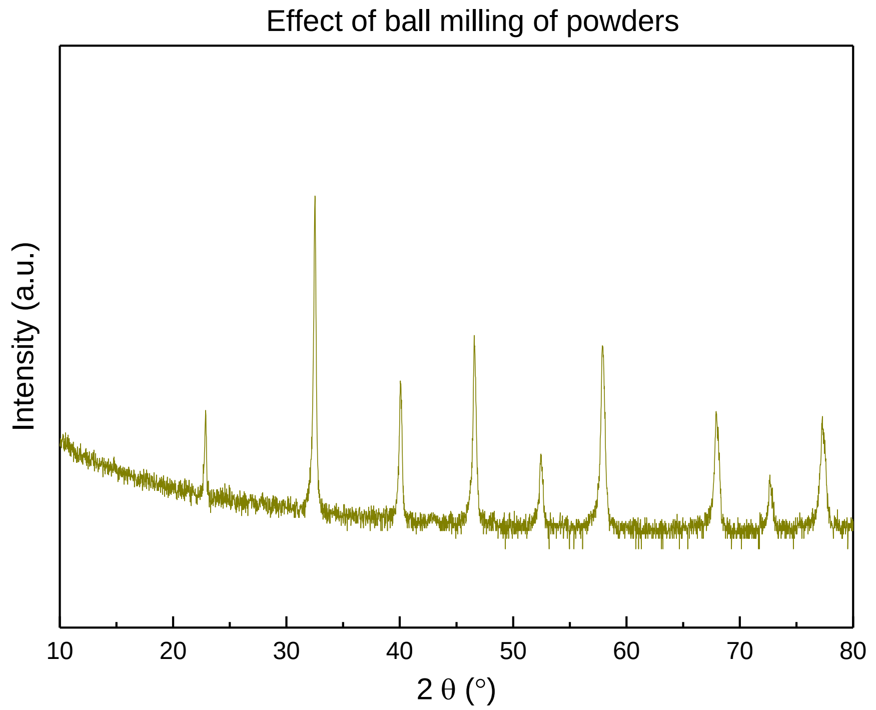

FIGURE S4 The XRD data of BLCF synthesized during investigation effect of ball milling of powders.
